# Visualization of Diagnostic and Therapeutic Targets in Glioma With Molecular Imaging

**DOI:** 10.3389/fimmu.2020.592389

**Published:** 2020-10-30

**Authors:** Deling Li, Chirag B. Patel, Guofan Xu, Andrei Iagaru, Zhaohui Zhu, Liwei Zhang, Zhen Cheng

**Affiliations:** ^1^ Department of Neurosurgery, Beijing Tiantan Hospital, Capital Medical University, China National Clinical Research Center for Neurological Diseases (NCRC-ND), Beijing, China; ^2^ Molecular Imaging Program at Stanford (MIPS), Department of Radiology, School of Medicine, Stanford University, Stanford, CA, United States; ^3^ Division of Neuro-Oncology, Department of Neurology and Neurological Sciences, School of Medicine, Stanford University, Stanford, CA, United States; ^4^ Department of Nuclear Medicine, Peking Union Medical College Hospital, Beijing, China; ^5^ Beijing Neurosurgical Institute, Capital Medical University, Beijing, China

**Keywords:** glioma, molecular imaging, probes, targeted therapy, precision medicine

## Abstract

Gliomas, particularly high-grade gliomas including glioblastoma (GBM), represent the most common and malignant types of primary brain cancer in adults, and carry a poor prognosis. GBM has been classified into distinct subgroups over the years based on cellular morphology, clinical characteristics, biomarkers, and neuroimaging findings. Based on these classifications, differences in therapeutic response and patient outcomes have been established. Recently, the identification of complex molecular signatures of GBM has led to the development of diverse targeted therapeutic regimens and translation into multiple clinical trials. Chemical-, peptide-, antibody-, and nanoparticle-based probes have been designed to target specific molecules in gliomas and then be visualized with multimodality molecular imaging (MI) techniques including positron emission tomography (PET), single-photon emission computed tomography (SPECT), near-infrared fluorescence (NIRF), bioluminescence imaging (BLI), and magnetic resonance imaging (MRI). Thus, multiple molecules of interest can now be noninvasively imaged to guide targeted therapies with a potential survival benefit. Here, we review developments in molecular-targeted diagnosis and therapy in glioma, MI of these targets, and MI monitoring of treatment response, with a focus on the biological mechanisms of these advanced molecular probes. MI probes have the potential to noninvasively demonstrate the pathophysiologic features of glioma for diagnostic, treatment, and response assessment considerations for various targeted therapies, including immunotherapy. However, most MI tracers are in preclinical development, with only integrin α_V_β_3_ and isocitrate dehydrogenase (IDH)-mutant MI tracers having been translated to patients. Expanded international collaborations would accelerate translational research in the field of glioma MI.

## Introduction

Gliomas, especially glioblastoma (GBM), are the most malignant primary brain tumors in adults (1). Numerous *in vitro*, *in vivo*, and *ex vivo* studies have revealed multiple molecular fingerprints of gliomas, such as methylation of the O(6)-methylguanine-DNA methyltransferase (MGMT) promoter, mutant isocitrate dehydrogenase (IDH), platelet-derived growth factor receptor (PDGFR), vascular endothelial growth factor receptor (VEGFR), integrin α_v_β_3_ receptor, epidermal growth factor receptor (EGFR), c-Met, etc. These tumor-specific molecules can be used not only as targets for diagnosis and therapeutic response assessment, but also as potential targets for glioma treatment. Recently, advances in techniques for identifying new molecules of interest and the rapid development of novel molecular targeted inhibitors have given rise to new molecular imaging (MI) agents that have been developed using this highly selective approach.

Developments in MI techniques enable the visualization, characterization, and measurement of biological processes at the molecular and cellular levels in living systems (2). MI probes are introduced noninvasively to determine the expression of molecular targets of interest in tumors and, when evaluated repeatedly over time in the same subject, enable the evaluation of tumor response to a given therapy. Considering the spatial and temporal heterogeneity are inherent in gliomas, MI can serve as a useful tool for overcoming some of the limitations of routine diagnostics. For example, although pathological diagnosis is considered the gold standard, it provides molecular characterization of the glioma at a single snapshot in time (e.g., prior to chemoradiation, or in the case of recurrent disease, after multiple treatments including chemoradiation) and is limited in scope to the tumor region sampled by neurosurgeon. In addition, multiple reports have demonstrated inter-rater variability for glioma pathology diagnosis among trained experts, and the superiority of molecular and genetic profiles compared to histological analyses for prediction of overall survival (OS) in patients with glioma (3, 4). Instead, by implementing an advanced MI-based approach, the molecular marker status of tumors could be interrogated repeatedly *in vivo* over the course of the patient’s treatment regimens. Accordingly, translational research involving these methods is currently underway at different stages including subcutaneous glioma animal models, orthotopic glioma animal models, and patients with glioma (e.g., NCT03539731).

Here, we searched PubMed (2000 to 2020) using the search terms “glioma” or “glioblastoma” in combination with “molecular imaging”, “positron emission tomography (PET)”, “fluorescence”, “magnetic resonance spectroscopy (MRS)”, and “single-photon emission computed tomography (SPECT)”. We included only articles published in English. The articles relevant to this topic were included for analysis. Next, we address the MI tracers developed for glioma and review their current stage of clinical translation. We also discuss nonspecific tracers (e.g., ^18^F-fluoro-2-deoxyglucose [^18^F-FDG] and radiolabeled amino acids) that are used to monitor for treatment response to anti-glioma therapies. Additional details about the tracers routinely utilized in glioma diagnosis and therapy have been reviewed previously (5–8). The goal of this review is to narrow the gap between multidisciplinary researchers in the fields of glioma molecular diagnosis, therapy, and imaging techniques, in order to ultimately help improve targeted diagnosis and therapy in glioma.

## Applications of Current Molecular Imaging Tracers in Targeted Therapy

In **Table 1**, we summarize distinct MI modalities, and their corresponding tracers, in the context of targeted therapies against glioma. Other advanced MR imaging (MRI) techniques such as MR perfusion imaging, dynamic susceptibility contrast (DSC) MRI, and diffusion-weighted MRI are summarized elsewhere (18, 19).

**Table 1:** Widely used nonspecific molecular imaging tracers to assess glioma response to targeted inhibitor therapies.

Probe	Article	Model for test	Molecule targeted	Agents	Key details of study
^18^F-FDG^1^	Graham et al. (9)	31 recurrent HGG patients	VEGF receptor	Bevacizumab	Prognostic of response to therapy and predictor of OS
^18^F-FDG and MRI^1^	Omuro A et al. (10)	40 newly diagnosed GBM patients	VEGF receptor	Bevacizumab and temozolomide	Higher baseline ADC ratios and persistent 6-month FDG-PET hypermetabolism predicted poor OS
^18^F-FET^1^	Fleischmann et al. (11)	72 recurrent HGG patients	VEGF receptor	Bevacizumab and re-irradiation	Minimal time-to-peak (TTPmin) provided a high prognostic value prior to re-irradiation
^18^F-FDOPA	Johannes et al. (12)	30 recurrent GBM patients	VEGF receptor	Bevacizumab	Identified treatment responders as early as two weeks after treatment initiation
^18^F-FDOPA	Robert et al. (13)	24 recurrent GBM patients	VEGF receptor	Bevacizumab	FDOPA or FLT PET uptake on parametric response maps after treatment as a useful biomarker for predicting PFS, FDOPA predicted patient OS
^18^F-FDG PET/MRI^1^	Benjamin et al. (14)	47 recurrent GBM patients	PI3-kinase and mTOR	GDC-0084	change in PET uptake, ADC, Ktrans, and relative cerebral blood volume correlated with maximum concentration of drug and PFS
^18^F-FLT, ^18^F-FET and MRI	Philip et al. (15)	U87MG (orthotopically in mice)	PI3-kinase and mTOR	Bevacizumab and BEZ235	More accurately predict the clinical potential with multimodality imaging
^18^F-FDG and ^18^F-FLT	Rex et al. (16)	U87MG (subcutaneously in mice)	c-Met	Rilotumumab and CE-355621	Accumulation of both radiotracers reduced as early as 2 and 4 days post-initiation of therapy
^18^F-FDG or ^18^F-FLT	Moonshi et al. (17)	U87MG (orthotopically in mice)	RTK	Sunitinib	Longitudinal ^18^F-FLT imaging detected therapeutic response at 7 days post-initiation of therapy, earlier than MRI (10 days) or ^18^F-FDG PET (16 days)

^1^Clinically used in glioma patients. ADC, apparent diffusion coefficient; c-Met, one cell surface receptor tyrosine kinase; HGG, high-grade glioma; FDG, fluorodeoxyglucose; FLT, fluorothymidine; FET, fluoro-ethyl-tyrosine; GBM, glioblastoma multiforme; MRI, magnetic resonance imaging; mTOR, mammalian target of rapamycin; OS, overall survival; PFS, progression-free survival; PI3, phosphoinositide 3-kinase; RTK, receptor tyrosine kinase; U87, human GBM cell line; VEGF, vascular endothelial growth factor.

The widely used oncologic and neurologic radiotracer, ^18^F-FDG, has been employed not only for evaluating the efficacy of bevacizumab [the only U.S. Food and Drug Administration (FDA)–approved targeted inhibitor for recurrent GBM (20)] for newly diagnosed and recurrent GBM (9, 10), but also for monitoring efficacy of novel inhibitors against molecular targets of interest in glioma, such as c-Met [a receptor tyrosine kinase (RTK) whose ligand is hepatocyte growth factor] (16), phosphoinositide 3 (PI3)-kinase (21), mammalian target of rapamycin (mTOR) (22), and other RTKs (17). These studies demonstrate that ^18^F-FDG PET/computed tomography (PET/CT) can potentially detect early metabolic changes that occur before alterations discernable on traditional anatomic MRI (e.g., tumor volume) and can thus help predict OS in these patients.

To evaluate the efficacy of novel targeted medications in glioma, other MI tracers besides ^18^F-FDG have been used. Goggi et al. compared various PET imaging radiotracers, including ^18^F-FDG, 3’-deoxy-3’-^18^F-fluorothymidine (^18^F-FLT), and 2-^18^F-fluoroethyl-triazolyl-conjugated c(RGDyK) peptide (^18^F-FtRGD), for early determination of tumor response to the antiangiogenic agent axitinib in mice bearing U87MG subcutaneous tumors (23). The results showed that the retention of ^18^F-FtRGD exhibited a much earlier attenuation in the tumor by Day 7 (Day 3 for ^18^F-FLT), compared to Day 10 for ^18^F-FDG. Moreover, a prospective study of 16 patients with recurrent high-grade glioma (HGG) treated with bevacizumab and irinotecan concluded that both ^18^F-FLT-avid and ^18^F-fluoro-ethyl-tyrosine (^18^F-FET)-avid volume reduction after two months of therapy predicted progression-free survival (PFS) and OS, and the volume-based analysis of ^18^F-FET uptake was superior to that of ^18^F-FLT in predicting patient survival (24).

^18^F-FLT PET has gained traction in neuro-oncology imaging in Europe to help guide targeted therapy for gliomas. The use of this probe allows for direct and correlated quantification of proliferation rates through expression of the enzyme thymidine kinase-1 during DNA synthesis at an early stage (25, 26). Other studies have evaluated the ^11^C-methyl-L-methionine (^11^C-Met) radiotracer, which has been demonstrated to be an early indicator, at 3 weeks, of tumor proliferation and vessel remodeling. By comparison, ^18^F-FLT uptake correlated with positive Ki-67 staining only at 6 weeks in an analysis of the dynamic growth of angiogenesis-dependent/independent experimental GBM (27). Compared to the 110-min half-life of ^18^F, the 20-min half-life of ^11^C makes the latter radioisotope less amenable to practical clinical translation.

In the United States, the more commonly used amino acid-based PET radiotracer is ^18^F-FDOPA and its uptake has been prospectively shown to be correlated with glioma grade and cellularity (28). A prospective study of 30 patients with recurrent HGG on bevacizumab therapy demonstrated that ^18^F-FDOPA PET identified treatment responders as early as two weeks after starting treatment (12). In an earlier study of ^18^F-FDOPA and ^18^F-FLT PET in recurrent HGG patients treated with bevacizumab, a post-treatment increase in uptake of both radiotracers on parametric response maps (PRMs) predicted PFS, but only the ^18^F-FDOPA PET PRMs predicted OS (13). One advantage of the amino acid-based tracers, including ^11^C-Met, ^18^F-FET, ^18^F-FLT and ^18^F-FDOPA, etc., is the fact that their uptake does not depend on blood-brain barrier (BBB) permeability.

In another study, patients treated with the indoleamine 2,3 dioxygenase 1 (IDO1) pathway inhibitor indoximod (D1-MT) and temozolomide underwent pre-treatment and on-treatment α-^11^C-methyl-L-tryptophan (AMT) PET, and post-treatment imaging showed decreased regional uptake of the radiotracer (29). Because IDO1 metabolizes tryptophan into kynurenine, this strategy of using AMT PET to monitor therapeutic response with an IDO1 inhibitor serves as an example of a PET radiotracer “companion diagnostic” to targeted molecular therapy in GBM.

## Molecules With Targeted Inhibitors Under Evaluation in Clinical Trials

Noninvasive imaging of the molecular events that occur in glioma has attracted increased research interest. Several promising molecular targets have been identified, including mutant IDH, PDGFR, VEGFR, integrin α_v_β_3_ receptor, EGFR, c-Met, etc., These molecules and their specific inhibitors have been studied in multiple trials, and we summarize the MI modalities that are being used to visualize them in the context of glioma therapy. With a focus on translation from pre-clinical models to human trials, relevant studies are summarized in **Table 2**.

**Table 2:** List of *in vivo* visualization of specific molecules whose targeted inhibitors are under evaluation in clinical trials.

Molecule	Article	Molecular imaging probes	Imaging instrument	Model for test	Key details of study	Targeted drugs
IDH mutation	Choi et al. (30)	None	3T Proton MRS	30 Glioma patients of all grades	Noninvasive detection of D-2HG	AGI-5198 (31), HMS-101 (32)
PDGFRβ	Tolmachev et al. (33)**^2^**	^111^In-DOTA-Z09591	SPECT/CT	U87MG (subcutaneous)		Imatinib, Dasatinib (34)
VEGFR2	He et al. (35)**^2^**	Anti-VEGFR2-albumin-Gd-DTPA	Molecular MRI	C6 or RG2 glioma-bearing rats (orthotopic)	Angiogenesis; intratumor and intertumor heterogeneity	Bevacizumab (20)
	Chen et al. (36)**^2^**	^64^Cu-DOTA-VEGF	PET	U87MG (subcutaneous in mice)	Quantitative; treatment monitoring	
	Rainer et al. (37)	^123^I-VEGF	SPECT	23 Glioma patients	Prognostic value for overall survival	
	Jansen et al. (38)	^89^Zr-Bevacizumab	PET	7 Children with diffuse intrinsic pontine glioma	Specific uptake in MRI contrast-enhanced areas, but with heterogeneous patterns	
Integrin α_v_β_3_	Iagaru et al. (39)	^18^F-FPPRGD2	PET	17 Recurrent GBM patients	Earlier identification of recurrence compared to MRI and ^18^F-FDG PET	Cilengitide (40);
	Li et al. (41)	^68^Ga-BNOTA- PRGD2	PET	12 Newly diagnosed glioma patients	Uptake correlated with grade	
	Schnell et al. (42)	^18^F-Galacto-RGD	PET	12 GBM patients (newly diagnosed and recurrent)	Significant but heterogeneous tracer uptake in microvessels and glial tumor cells	
	Lee et al. (43)**^2^**	RGD- NaGdF4:Yb3+/Er3+ nanophosphor	PET and 3T T1-weighted MRI	U87MG (subcutaneous in mice)		
	Morales-Avila et al. (44) **^2^**	^99m^Tc-HYNIC-GGC-AuNP-c[RGDfK(C)	Micro-SPECT/CT	C6-Induced tumors with blocked/nonblocked receptors (subcutaneous in mice)		
	Lanzardo et al. (45) **^2^**	RGD cyclic probe (DA364)	NIRF	U87MG (subcutaneous in mice)		
	Hsu et al. (46) **^2^**	Cy5.5-linked cyclic RGD peptide	NIRF and BLI	U87MG expressing luciferase (orthotopic in mice)	Angiogenesis	
	Ellegala et al. (47) **^2^**		PET	U87MG (orthotopic in mice)	Biodistribution of tracer and MET expression	
	Choi et al. (48) **^2^**	^123^I- and ^68^Ga- RGD-HSA-TIMP2	SPECT and PET	U87MG (subcutaneous in mice)	TIMP2 as an inhibitor of angiogenesis, also targets integrin α_v_β_3_	
Integrin α_v_β_3_ and TIMP2	Tang et al. (49) **^2^**	^89^Zr-DFO-nimotuzumab	PET	U87MG expressing EGFR (subcutaneous in mice)	Assessing EGFR status	
EGFRvIII	Elliott et al. (50) **^2^**	ABY-029	NIRF	F98 expressing EGFR (orthotopic in mice)	Outperformed 5-ALA for fluorescence-guided surgery in EGFR+ tumors	Erlotinib (51); EGFR-retargeted oncolytic herpes simplex virus (mice) (52) CDX-110 (53) CAR-modified T (CART)-EGFRvIII cells (54)
	Fatehi et al. (55) **^2^**	Qd800 to an anti-EGFRvIII single domain antibody (EG2-Cys)	NIRF	U87MG (subcutaneous in mice)	Correlated with aggressiveness and resistance	
	Mishra et al. (56) **^2^**	EGFR conjugated metal chelates	SPECT	U-87MG and MDA-MB-468 (subcutaneous in mice)		
	Davis et al. (57) **^2^**	Gadolinium contrast; near-infrared fluorophore bound to EGF ligand	MRI-coupled FMT	U251 and 9L-GFP (orthotopic in mice)	Quantification of EGFR receptor	
	Zhang et al. (58) **^2^**	Engineered Bioluminescence Met reporter (BMR)	BLI	U87MG (subcutaneous in mice)	Pharmacokinetics and bioavailability of c-Met specific agents	
c-Met	Terwisscha et al. (59) **^2^**	Anticalin ^89^Zr-PRS-110	PET	U87MG (subcutaneous in mice)	Specific uptake and earlier accumulation in c-Met-expressing tumors	AMG102 (60) Crizotinib (61)
	Jun et al. (62) **^2^**	None	BLI	c-MET-positive and c-MET-negative luciferase-expressing primary GBM tumor cells (orthotopic in mice)	Correlating c-Met expression status with tumor growth	
	Kim et al. (63) **^2^**	^125^I-labeled MET-binding peptides	SPECT/CT	U87MG (subcutaneous in mice)	Visualizing tumor but with unremarkable overall image quality	
	Jagoda et al. (64) **^2^**	^89^Zr-df-Onartuzum vs. ^76^Br-Onartuzumab	PET	U87MG (subcutaneous in mice)	Improved c-Met imaging for prognostic purposes	

^2^Only in vivo imaging including glioma patients and animal model, but excluding in vitro imaging. 5-ALA, 5-aminolevulinic acid; 9L-GFP, rat gliosarcoma cell line expressing GFP; α_v_β_3_, alpha(V) beta(3); BLI, bioluminescence imaging; c-Met, tyrosine-protein kinase Met or hepatocyte growth factor receptor; CT, computed tomography; D-2HG, D-2-hydroxyglutarate; DOTA, tetraxetan; DFO, desferoxamine; EGFR, epidermal growth factor receptor; FDG, fluorodeoxyglucose; FMT, fluorescence molecular tomography; GBM, glioblastoma multiforme; Gd-DTPA, gadolinium with diethylenetriaminepentacetate; HSA, human serum albumin; MRI, magnetic resonance imaging; MRS, magnetic resonance spectroscopy; NIRF, near-infrared fluorescence; NOTA, 1,4,7-triazacyclononane-1,4,7-triacetic acid; PDGF, platelet-derived growth factor; PET, positron emission tomography; RGD, tripeptide Arg–Gly–Asp; SPECT, single-photon emission computed tomography; TIMP, tissue inhibitor of metalloproteinase; U87, human GBM cell line; VEGF, vascular endothelial growth factor; U251, human GBM cell line.

## IDH Mutation and Its Inhibitors

IDH mutation was identified in most astrocytomas and secondary GBM as an early and inducing event in gliomagenesis (65, 66). IDH mutation status is a predictive marker of the therapeutic efficacy of alkylating chemotherapy in HGG patients (67, 68) and has also been associated with improved prognostic (i.e., OS) value in HGG and low-grade glioma (LGG) (65, 69). Therefore, IDH mutational status was introduced into the 2016 World Health Organization (WHO) classification of cancers of the central nervous system as a crucial molecular genetic feature (70). In addition, the presence of IDH mutation itself represents a therapeutic target in glioma, and several IDH1 mutation inhibitors have been evaluated in IDH-mutant glioma patients (71).

IDH mutation can be detected using various *ex vivo* methods, including direct sequencing (65, 72), allele-specific PCR (73), and immunohistochemistry (IHC) (74). Several studies have also focused on D-2-hydroxyglutarate (D-2HG). Santagata et al. used desorption electrospray-ionization mass spectrometry to detect D-2HG *ex vivo* and found that its signal overlaps with areas of tumor and correlates with the tumor contents. They further suggested that mapping the D-2HG signal onto anatomic 3D reconstructed MR images of tumors can be integrated with advanced multimodality image-guided neurosurgical procedures to enable rapid molecular analysis of surgical tissue intraoperatively (75).

**Figure 1:**
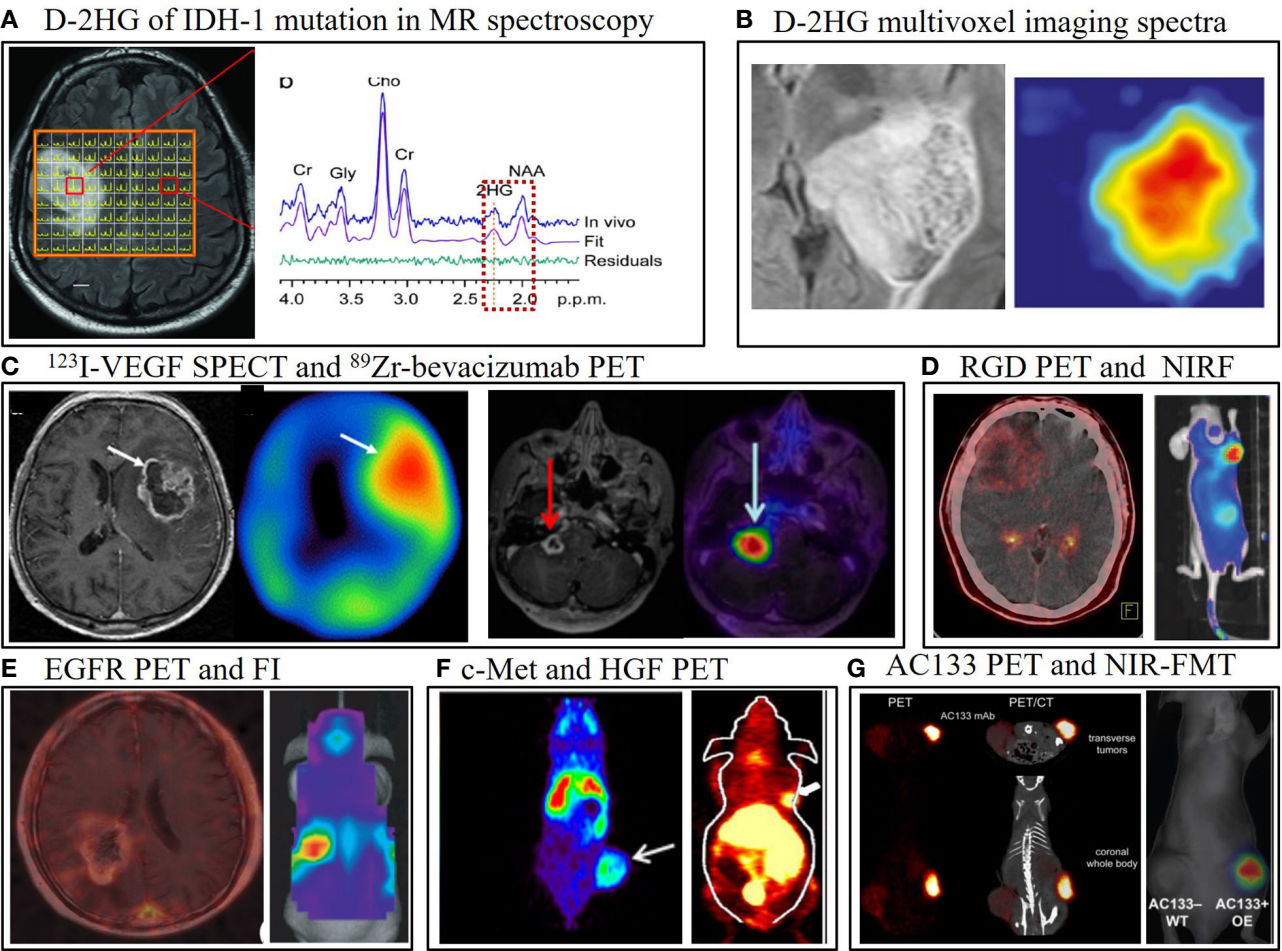
Representative multimodality molecular imaging in glioma, including positron emission tomography (PET), single-photon emission computed tomography (SPECT), optical, and MR spectroscopy (MRS). **(A)** The major catabolite of IDH-1 mutation in gliomas, D-2-hydroxyglutarate (D-2HG), can be visualized by MRS, and this technique has been translated to clinical trials (30). **(B)** T2/FLAIR abnormal signal area in MRI is overlaid with the D-2HG multivoxel imaging spectra in MRS (76). **(C)** Glioblastoma lesion uptake with the ^123^I-VEGF SPECT tracer (left) (37) and the ^89^Zr-bevacizumab PET radiotracer (144 h post-injection) fused with gadolinium-enhanced T1-weighted MRI in a child with diffuse intrinsic pontine glioma (right) (38). **(D)** Integrin α_v_β_3_ visualized in a patient with glioblastoma using ^68^Ga-PRGD2 PET/CT by our team; RGD-Cy5.5 conjugate near-infrared fluorescence (NIRF) image showing integrin α_v_β_3_ in a mouse bearing a subcutaneous U87MG tumor (77). **(E)** ^11^C-PD153035 PET/CT for visualization of EGFR in human glioblastoma (78); *in vivo* optical imaging of epidermal growth factor receptor variant III (EGFRvIII)-expressing U87MG cells orthotopically implanted in a mouse identifies the tumor after intravenous injection of a EGFRvIII single-domain antibody bioconjugated to near-infrared quantum dots, with an extra cysteine for site-specific conjugation (55). **(F)** ^89^Zr-PRS-110 PET noninvasively shows c-Met positivity in a U87MG subcutaneous tumor model (59). ^64^Cu-labeled recombinant human hepatocyte growth factor PET also detects c-Met expression in nude mice bearing U87MG xenografted tumors (79). **(G)** Mouse bearing AC133/CD133-overexpressing U251 gliomas in a subcutaneous tumor model can be imaged with ^64^Cu-NOTA-AC133 mAb PET/CT (80); IR700-conjugated AC133 can also identify the tumor using near-infrared fluorescence (NIRF) molecular tomography (FMT) (81). All images have been reprinted with permission; **(D)** is previously unpublished data.

*In vivo* imaging of IDH mutation has attracted considerable attention. However, because of the technical challenges associated with imaging the gene mutation itself, the MI approaches are currently based on D-2HG. Choi et al. estimated the concentration of D-2HG by performing spectral fitting in the case of tumors from 30 patients. Numerical and phantom analyses of MRS pulse sequences were performed, and the results were validated with mass spectrometry of *ex vivo* tissues and then successfully translated to clinic with a larger prospective trial (30, 76). Such *in vivo* MRS methods have also been shown to detect IDH mutations (**Figures 1A, B**) that were missed in IHC analyses, and the reduction in D-2HG levels has been used to monitor treatment response in patients with IDH-mutant gliomas and correlated with clinical status (82, 83). A recent clinical trial and pooled analysis demonstrated the high sensitivity and specificity of MRS compared to other imaging modalities for the detection of IDH mutational status (84, 85). MRS was used to serially monitor for a decrement of D-2HG levels in gliomas in a Phase I clinical trial of a new mutant IDH1 inhibitor (86). To date, no specific IDH-mutant-specific targeted MI probe has been developed for PET or SPECT. Nonspecific probes such as ^18^F-FDOPA were shown to accumulate in LGG with IDH mutation (87). A more recent study suggests that dynamic ^18^F-FDOPA uptake parameters (e.g., time to peak SUV) rather than static uptake parameters (e.g., SUVmax) may be able to discriminate between IDH mutant and IDH wild-type gliomas (88).

MI of D-2HG as a marker of IDH mutant status by MRS has achieved successful clinical translation in glioma patients and can be used to serially and noninvasively monitor for this important pathophysiologic molecular marker. Further research should be conducted to integrate this imaging modality as a neuroimaging “companion diagnostic” in clinical trials of therapies targeting the IDH1 mutation, to determine whether it can stratify patients into the responder and non-responder subsets. More novel MI techniques with higher sensitivity, higher specificity, and lower dependence on BBB permeability should be developed, in light of the low sensitivity of MRS for detecting IDH mutant status in smaller tumors due to partial-volume effects (89).

## PDGFR and Src Family Kinases (SFKs) and Their Inhibitors

PDGFR plays a critical role in HGG and synergizes with SFKs, which are nonreceptor membrane-associated tyrosine kinases. PDGFR and SFKs are both associated with the invasiveness (90), self-renewal of glioma-initiating cells, and growth of tumor vasculature in HGG (91). PDGFRβ is expressed not only in vasculature, but also in GBM-associated stromal cells, which exert tumor-promoting effects on glioma cells *in vitro* and *in vivo* (92).

Specific targeted inhibitors of PDGFRβ include first-generation single-kinase inhibitors (e.g., imatinib) and second-generation inhibitors of multiple protein tyrosine kinases (e.g., dasatinib, which targets both PDGFR and SFKs). Dasatinib has been shown to inhibit bevacizumab-induced glioma cell invasion in an orthotopic xenograft model, supporting the human translation of combining dasatinib with bevacizumab in HGG (93). However, recent clinical trials showed that dasatinib in conjunction with bevacizumab did not appear to benefit patients with newly diagnosed and recurrent GBM (94, 95). MEDI-575, an immunoglobulin G2κ monoclonal antibody that selectively binds to platelet-derived growth factor receptor α (PDGFRα), also showed limited clinical efficacy in recurrent GBM in a Phase II clinical trial (96).

Developments in visualizing PDGFR expression in glioma *via* MI are relatively insufficient. Tolmachev et al. designed a PDGFRβ-binding affibody molecule, Z09591, which was labeled with ^111^In to specifically visualize PDGFRβ expression; the affibody was used for imaging in an U87MG xenograft model by applying small-animal SPECT/CT (33). Future studies of novel PET radiotracers are warranted because they may provide increased sensitivity, specificity, and quantification accuracy. In conclusion, PDGFR can be used as a pathophysiologic marker of glioma but much work still remains for further PDGFR-based targeted therapy and imaging.

## VEGFR and Bevacizumab

VEGF is the key pro-angiogenic protein that is overexpressed in and released by gliomas into their microenvironment (97). Glioma treatment with bevacizumab, an inhibitor of VEGF receptor (VEGFR) expressed on vascular endothelium, has led to increased PFS but no OS benefit in the patients with recurrent GBM and was approved for GBM therapy in 2009 (98). However, bevacizumab failed to show a survival advantage in two large studies of patients with newly diagnosed GBM: AVaglio in Europe and RTOG-0825 in North America (99, 100).

Selecting appropriate candidates for optimal antiangiogenic therapy is critical, and this has recently attracted considerable research attention. EGFR gene amplification are associated with shorter time to progression in patients with recurrent GBM while treated with bevacizumab (101). Other tissue-based and advanced neuroimaging parameters that are used as potential biomarkers in the setting of anti-VEGFR therapy are reviewed elsewhere (102). The ^18^F-radiolabeled FET, FLT, and FDG PET tracers mentioned earlier are based on cell proliferation and metabolism and can be used to indirectly assess anti-VEGFR treatment response (103). Here, we focus on VEGFR-specific MI, which may help in identifying suitable candidates for antiangiogenic treatment, as well as in evaluating treatment response and disease progression. An anti-VEGFR probe (anti-VEGFR-albumin-gadolinium) was designed to image VEGFR in C6 and RG2 glioma-bearing rats with MRI, and the findings were further confirmed through fluorescence staining and quantification of the fluorescence intensity of the anti-VEGFR probe (35). Moreover, a PET tracer, ^64^Cu-DOTA-VEGF, was developed for use in small-animal PET to quantify VEGFR expression levels in animal models *in vivo* (36).A clinical research demonstrated the SPECT using recombinant human VEGF labeled with ^123^I can visualize GBM rather than LGG and stratify patients’ OS based on specific T/N ratio threshold (37) (**Figure 1C**, left). In HGG, VEGF-based radiotracer approaches used to assess response to therapy may be confounded by endogenous VEGF levels in the tumor microenvironment that compete to bind for the same VEGFR’s on the vascular endothelium. Therefore, another approach would be to develop an anti-VEGFR-based radiolabeled antibody. An immunoPET tracer, ^89^Zr-bevacizumab, was designed using a diagnostic radioisotope with the commercial antibody drug (Avastin^®^) to visualize the heterogeneity of binding of this drug on the vascular endothelium in diffuse intrinsic pontine glioma (DIPG) (38) (**Figure 1C**, right).

In conclusion, VEGFR has been successfully targeted with bevacizumab as an approved therapy for recurrent GBM, and its effects could be monitored with several MI techniques. Further investigation is required to correlate these VEGF- and VEGFR-targeted MI techniques with treatment efficacy in clinical trials of bevacizumab therapy for GBM, which has potential to identify the patient subset that is most likely to respond to therapy. Taking the relatively large molecular weights of VEGF or antibody into consideration, the BBB influence of these tracers should be investigated further. The newer anti-angiogenic agents in GBM, e.g., anti-VEGF therapies like TTAC-0001 (NCT03856099), could similarly be evaluated with this MI-based approach.

## Integrin α_V_β_3_ and Cilengitide

Integrin alpha(V)beta(3) (α_v_β_3_) was shown to be overexpressed in neogenic vessels and glioma cells *in vitro* (104) and *ex vivo* (105); the expression of this integrin generally correlates with malignancy grade and is a negative prognostic factor (105). Several inhibitors targeting integrin α_v_β_3_ are under development. Cilengitide, a selective α_V_β_3_ and α_V_β_5_ integrin inhibitor, has been shown to inhibit GBM growth in preclinical tumor models, as well as in patients with newly diagnosed and recurrent GBM in Phase I and II clinical trials (106–110). However, in the Phase III CENTRIC EORTC 26071-22072 trial, Stupp et al. reported no OS benefit when this inhibitor was combined with standard chemotherapy in newly diagnosed GBM patients with methylation of the MGMT promoter (111).

Chinot noted several possible reasons for the failure of that trial, including screening based on MGMT promoter methylation status when this biomarker may not necessarily be associated with integrin biology (112). Another reason for failure of that trial may be the heterogeneity of integrin α_v_β_3_ expression in GBM, which was clearly demonstrated by *ex vivo* IHC (105) and *in vivo* MI studies (42). Targeted therapy is likely to be effective only when the defined target molecule is expressed at high levels. Thus, for GBM treatment, a rational MI-based approach for future clinical trials would be to (1) confirm the existence of the target as a screening inclusion criterion before initiating integrin-inhibitor treatment and (2) serially track expression of the molecular target as a physiologic surrogate for monitoring tumor response alongside traditional anatomic MRI.

Noninvasive visualization of integrins in the setting of cancer has been developed over the past decades. Sipkins et al. visualized integrin α_v_β_3_ by using Gd-containing liposomes coated with a monoclonal antibody (mAb) in animal models of breast cancer and malignant melanoma (113). Integrin imaging for several tumor types *via* multimodality imaging including MRI, ultrasound, near-infrared fluorescence (NIRF) imaging, SPECT, and PET has been reviewed elsewhere (114).

NIRF dyes conjugated with a cyclic arginine-glycine-aspartic acid (RGD) peptide were applied to visualize subcutaneously inoculated integrin-positive gliomas (46, 77, 115). Chen et al. confirmed that the specific RGD peptide−integrin interaction which was detected using the NIRF technique could be employed to noninvasively image integrin expression in almost real-time in U87MG GBM xenografts (**Figure 1D**, right) (77). A study using ^64^Cu-cyclam-RAFT-c(-RGDfK-)4 in a mouse model of glioma demonstrated its therapeutic efficacy and suitability for integrin imaging in the tumor (116).

The RGD-based MI tracers and techniques have been successfully translated to patients in clinical trials. ^18^F-FPPRGD2, an RGD-dimer PET tracer, was evaluated for imaging the expression of integrin α_v_β_3_ in healthy volunteers and in patients with GBM and other cancers requiring antiangiogenic treatment (117). ^18^F-galacto-RGD was found to have marked yet heterogeneous uptake in microvessels and glial tumor cells (42). In another study, a relatively more specific dimer, ^68^Ga-BNOTA-PRGD2, was utilized (**Figure 1D**, left) and a semiquantitative feature of uptake was correlated with tumor grade (41). A clinical study of ^18^F-AlF-NOTA-PRGD2 PET/CT in newly diagnosed GBM patients showed that this integrin-targeting PET approach predicted response to chemoradiation (84.6% sensitivity, 90.0% specificity, and 87.0% accuracy) as early as 3 weeks post-initiation of treatment when using a SUVmax threshold of 1.35 (118). How much these typical peptide-based imaging tracers depend on BBB breakdown for imaging have not thoroughly assessed in suitable models.

Although integrin α_v_β_3_-targeted inhibitors were effective in preclinical studies and small cohorts of GBM patients in phase I and II clinical trials, they failed to demonstrate a survival benefit in a Phase III trial. However, integrin receptor imaging has been successfully translated to small pilot clinical studies of GBM patients and can be used to noninvasively demonstrate the integrin receptor distribution and expression density, which supports its use as a predictive neuroimaging biomarker during screening for prospective trial participants. Before this imaging can become a reliable predictive indicator for a specific subgroup of glioma patients, the imaging probes and techniques should be further validated for improved sensitivity and specificity in human patients.

## EGFR and Its Inhibitors

EGFR gene amplification and overexpression are striking features of GBM, particularly primary GBM. In approximately 50% of tumors showing EGFR amplification, a specific EGFR mutant, EGFR variant III (EGFRvIII), can be detected. EGFRvIII is specifically expressed in 31% of primary GBM patients, and compared to patients with wild-type EGFR GBM, those with EGFR-mutant GBM tend to have an older age at diagnosis, worse prognosis, and resistance to chemoradiotherapy (119, 120).

In addition to EGFR inhibitors (e.g., erlotinib), oncolytic HSV retargeted to GBM EGFR (52) and EGFRvIII vaccines have been evaluated in clinical trials. Rindopepimut (CDX-110) was designed to generate a specific immune response against EGFRvIII-expressing tumors, and the drug was demonstrated to benefit EGFRvIII-positive GBM patients in a Phase II trial, although it failed in a Phase III trial (ACT IV) of newly diagnosed, EGFRvIII-positive GBM patients (121, 122). Binder and colleagues reviewed possible reasons for failure of that trial, including loss of GBM EGFRvIII expression in ~60% of cases regardless of whether rindopepimut or control treatment was administered, and the lack of control arms in the previous promising Phase II trials (123). The incorporation of MI in such clinical trials to non-invasively detect the loss of expression of the target protein could prompt an earlier determination of lack of treatment efficacy, so a new therapy could be initiated that may lead to improved patient outcomes.

The first-in-human study of the chimeric antigen receptor modified T cell (CART)-EGFRvIII, as a cellular immunotherapy, in 10 recurrent GBM patients demonstrated on-target activity in brain. One patient had stable disease for over 18 months. However, the investigators found that the antigen expression decreased in the biopsied tissue in most patients (54). We believe that MI of antigen heterogeneity and reductions in antigen expression may provide earlier detection that the current therapy may no longer be efficacious, so that a different therapeutic strategy can be pursued earlier on.

EGFR-specific tracers were developed for multiple imaging modalities including SPECT, optical imaging, and MRI. Mishra et al. used anti-EGFR antibody-conjugated metal chelates in SPECT to image EGFR expression in mice bearing glioma cell lines (56). In another study, near-infrared imaging was performed on mice bearing orthotopic GBM by using a method in which a near-infrared quantum dot (Qd800) was conjugated to an anti-EGFRvIII single-domain (sd) antibody containing an extra cysteine to enable site-specific conjugation (EG2-Cys) (**Figure 1E**, right); this quantum dot-modified probe showed increased accumulation in tumors relative to the unconjugated quantum dot or the quantum dot conjugated to the Fc region of the antibody (EG2-hFc) (55). Another specific NIRF tracer, ABY-029, outperformed 5-ALA in detecting the tumor margin of EGFR-positive tumors and has the potential to enhance fluorescence-guided surgery (50). Lastly, ^11^C-PD153035 PET/CT was demonstrated to be positively correlated with *ex vivo* EGFR immunostaining and Western blot analysis in the case of glioma patients (**Figure 1E**, left) (78).

Davis et al. designed a MRI-coupled fluorescence molecular tomography (FMT) system in which gadolinium (Gd)–based contrast was used and a near-infrared fluorophore was bound to EGF, the ligand of EGFR. By using this system, the EGFR expression status in animal models of U251 and 9L-GFP tumors was quantified with 100% sensitivity and specificity (57). The FMT system was particularly effective when used in combination with the anatomy-based information provided by the Gd-enhanced MRI scan data.

Therefore, specific types of EGFR mutations should be screened with MI probes to investigate their utilization as imaging biomarkers for selecting patients for oncologic vaccine-based approaches. Future studies should also examine whether targeted EGFR-mutant MI tracers can be used to direct EGFR-targeted therapy *in vivo*.

## c-Met and Its Inhibitors

Hepatocyte growth factor/scatter factor (HGF/SF) and its cell-surface receptor, the tyrosine kinase c-Met, were found to be closely linked with glioma cell invasion and tumor progression (124), and c-Met has been widely confirmed as a crucial predictor of GBM patient outcomes (125).

Nearly two decades ago, c-Met expression was not only demonstrated in glioma cells and tumor microvasculature, but was also shown to be associated with astrocytic tumors through immunohistochemical staining of *ex vivo* glioma samples. Elevated c-Met expression levels paralleled higher tumor grades: 21.4% positive in astrocytoma (WHO grade II) and 53.8% positive in anaplastic astrocytoma as compared with 87.5% in GBM (126). Moreover, recent research has demonstrated increased efficacy of a prognosis model that includes c-Met protein expression (127). Jun et al. found c-Met was preferentially localized in the perivascular regions of human GBM tissues that are highly clonogenic, tumorigenic, and resistant to radiation. Bioluminescence imaging (BLI) was used to monitor tumor growth in nude mouse brains implanted with c-Met-positive and c-Met-negative luciferase-expressing GBM tumor cells, and this confirmed the relationship between c-Met expression tumor growth *in vivo* (62).

Both c-Met pathway-targeting small molecules and mAbs have been investigated in GBM, yielding promising results. AMG 102 (rilotumumab) enhanced the efficacy of temozolomide or docetaxel in U87MG cells and xenografts (60). However, in a Phase II clinical trial of rilotumumab in heavily pretreated patients with recurrent GBM, monotherapy was not associated with significant antitumor activity (128). Cabozantinib (XL184), an oral inhibitor of multiple RTKs such as c-Met and VEGFR2, yielded favorable results in the case of advanced prostate cancer (129), thyroid cancer (130), and was approved by the U.S. FDA in 2012. Interestingly, the Phase II trial of XL184 in recurrent GBM demonstrated antitumor activity, particularly in the antiangiogenic treatment-naive cohort, with a median PFS of 3.7 months in both the 140 mg/day and 100 mg/day groups (131). In the subset of patients who had received prior antiangiogenic therapy, the objective response rate was only 4.3% with a median duration of response of 4.2 months (132).

Knockdown of the c-Met protein can make tumor necrosis factor related apoptosis-inducing ligand (TRAIL)-resistant brain tumor cells sensitive to TRAIL treatment *in vitro*; moreover, in nude mice intracerebrally implanted with a c-Met-knockdown tumor cell line, the effect of stem cell-delivered S-TRAIL *in vivo* was confirmed using BLI (133). Zhang et al. monitored gene expression quantitatively and dynamically in cultured cells and in a U87MG tumor xenograft model by using a genetically engineered bioluminescent c-Met reporter gene (58). This novel MI of the reporter gene has been gradually used to visualize the crosstalk among different relevant molecular targets in glioma animal models.

Several groups have developed new radionuclide tracers to image c-Met expression in gliomas *in vivo*. With SPECT imaging, the tumor can be visualized using ^125^I-labeled c-Met-binding peptides in human U87MG tumor-bearing mice (63). Onartuzumab, an experimental therapeutic anti-c-Met mAb, was radiolabeled with ^76^Br or ^89^Zr, and the resulting probes showed minimal background in normal brain (64). Terwisscha van Scheltinga et al. visualized c-Met expression by using an anticalin ^89^Zr-PRS-110 PET radiotracer in U87MG xenografts (**Figure 1F**, left); however, nearly 40% nonspecific uptake of this probe was confirmed in the blocking experiment, and thus further investigation is necessary (59). In another study, recombinant human HGF was labeled with ^64^Cu, and this probe had strong and specific binding to c-Met in a U87MG tumor model (**Figure 1F**, right) (79).

In summary, all the MI techniques for visualizing c-Met expression are in the preclinical phase, and they will be clinically translated after the development of targeted drugs evaluated in clinical trials.

## Visualization of Specific Molecules That Do Not Yet Have Inhibitors Under Evaluation in Clinical Trials

In addition to the molecular targets for diagnosis, treatment, and imaging, other molecules exist that better characterize glioma pathophysiology including glioma stem-like cells, newly formed tumor blood vessels, etc. However, specific inhibitors against these emerging molecular biomarkers have not yet been evaluated in clinical trials. The relevant studies are summarized in **Table 3**.

**Table 3:** List of *in vivo* visualization of specific molecules that do not yet have inhibitors under evaluation in clinical trials.

Molecule	Article	Utilized imaging probes	Imaging modality	Model for test	Key details of study
CD133	Gaedicke et al. (80)	^64^Cu-NOTA-AC133 mAb	MicroPET	Orthotopic glioma xenografts (subcutaneous)	Monitoring of AC133(+) glioblastoma stem cells
	Jing H et al. (81)	IR700-AC133 mAb	NIRF	Orthotopic gliomas (subcutaneous)	Non-invasive detection of AC133 and linked with photoimmunotherapy
ELTD1	Towner et al. (134)	Anti-ELTD1 SPIO-based probe	Molecular MRI	F98 (orthotopic in rat)	Signal correlated with grade and survival

CD133, promonin-1; ELTD1, epidermal growth factor, latrophilin, and 7 transmembrane domain-containing protein 1 on chromosome 1; F98, rat GBM cell line; mAb, monoclonal antibody; NIRF, near-infrared fluorescence; NOTA, 1,4,7-triazacyclononane-1,4,7-triacetic acid; PET, positron emission tomography; SPIO, superparamagnetic iron oxide.

## CD133 and Glioma Stem Cells

Glioma cancer stem cells (CSCs) are resistant to chemoradiotherapy and have attracted the attention of multidisciplinary researchers. Gaedicke et al. developed a new imaging tracer targeting the AC133 epitope of CD133, which is a well-investigated CSC marker. An AC133-specific mAb was radiolabeled with ^64^Cu to generate ^64^Cu-NOTA-AC133 mAb, which was used to monitor AC133-positive GBM CSCs. High-sensitivity and high-resolution images were obtained in animal models using both PET and NIRF imaging (**Figure 1G**) (80). A novel small peptide, CBP4, was linked to gold nanoparticles and the resultant probe was shown to be suitable as an imaging agent for CD133-expressing GBM CSCs (135). Jing et al. conjugated the AC133 antibody with an IR700 dye and showed that the resulting probe can be used noninvasively to assess AC133-positive gliomas *via* near-infrared FMT; the probe was employed in near-infrared photoimmunotherapy to effectively induce cell death and tumor shrinkage in an animal model (81).

## ELTD1

EGF, latrophilin, and 7-transmembrane domain-containing protein 1 on chromosome 1 (ELTD1) was identified as a putative glioma-associated marker using a bioinformatics method and reported to be associated with glioma grade and patient survival by Towner et al. (134). An anti-ELTD1 superparamagnetic iron oxide (SPIO)-based probe was designed by coating SPIO nanoparticles with dextran and conjugating an anti-ELTD1 antibody. This probe was used to assess the *in vivo* levels of ELTD1, and further investigation revealed that the anti-ELTD1 antibody inhibited glioma growth in mouse glioma models, an effect that could be attributed to diminished vascularization (136).

## Progress in Clinical Translation of Various Tracers With Different Molecular Imaging Techniques

**Figure 2:**
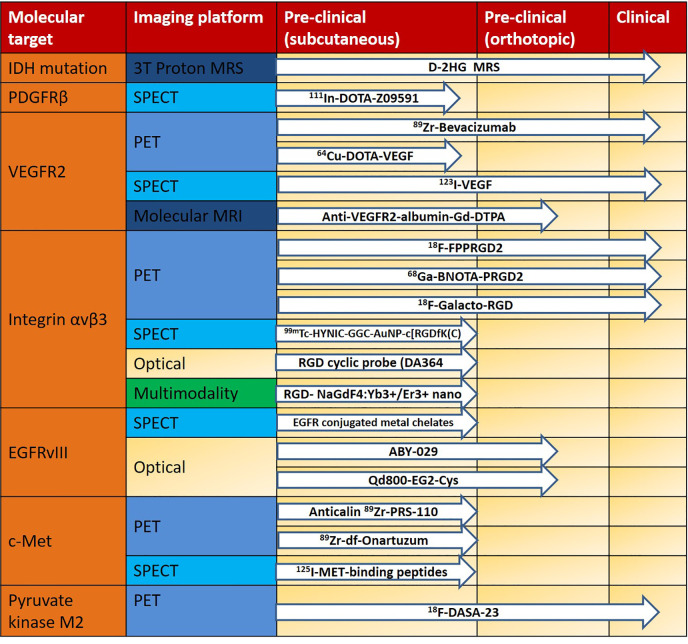
Translational pipeline of molecular imaging probes in glioma using different imaging platforms. IDH, isocitrate dehydrogenase; MRS, magnetic resonance spectroscopy; PDGFRβ, platelet-derived growth factor receptor beta; SPECT, single-photon emission computed tomography; VEGFR2, vascular endothelial growth factor receptor 2; PET, positron emission tomography; MRI, magnetic resonance imaging; Integrin αvβ3, integrin alpha(V)beta(3); EGFRvIII, epidermal growth factor receptor variant III.

We divided the translation process (from bench to bedside) into three stages of development: (1) Preclinical stage that includes subcutaneous animal models with glioma cell lines; (2) Preclinical stage that includes orthotopic animal models with glioma cell lines; and (3) Clinical stage that involves glioma patients. In **Figure 2**, we summarize the progress from pre-clinical to clinical translation of the abovementioned targeted MI tracers. Most of the targeted tracers have only been studied in animal models. The MI studies evaluated in human glioma patients target integrin α_V_β_3_, IDH-mutation and VEGFR, pyruvate kinase M2 and have been imaged using PET/CT, SPECT and MRI modalities. The superior molecular sensitivity of PET, the lack of radiation, and high spatial resolution of MRI render these techniques much easier to translate, along with the fact that they are routinely used in the medical field. Optical imaging (e.g., NIRF and BLI), have also been utilized to image molecular expression in glioma xenografts in subcutaneous and orthotopic animal models. Although penetration depth remains a challenge in optical imaging, intraoperative imaging could represent a promising area of research following further development in both imaging technique and tracer design. Multimodality imaging can provide a possible solution to overcome certain limitations of current methods (e.g., PET and MRI for imaging integrin α_v_β_3_, or optical imaging and MRI for imaging EGFR and IGFBP7). This strategy could enable imaging to be performed, using a single probe, on multiple imaging platforms with diverse disease models, ranging from small animal models to large animal models and even humans.

## Conclusions and Perspectives

With the discovery of multiple new molecular targets in glioma, the design and clinical translation of novel targeted diagnostics, treatments, and MI techniques have rapidly developed. MI offers several promising advantages over conventional anatomic imaging in glioma. Firstly, specific molecular expression patterns and therapeutic responses can be serially imaged *in vivo*, particularly for HGG patients, who typically undergo surgical treatment once at the time of initial diagnosis. Because of the minimal risk to patients, MI can be performed repeatedly if necessary, and can be used to evaluate tumor heterogeneity across the entire tumor, including its resected and residual components. Secondly, MI can potentially visualize prognostic and predictive biomarkers of interest to aid in selecting appropriate patients for molecular-targeted therapy. This approach would promote the evidence-based selection of patients for molecular-targeted therapeutic clinical trials and thereby possibly increase the success of improving survival in the appropriate patient cohort. Thirdly, MI can be applied routinely for the development and assessment of novel anti-glioma drugs or immunotherapy agents, because it can accurately monitor the pharmacodynamic and bioavailability of therapeutics in tumors.

Multimodality imaging probes can be designed to detect multiple biomarkers concurrently in glioma patients, and thus noninvasively map crucial molecules in this heterogenous and challenging disease. Given the advantages mentioned above, MI can represent an optimal method for achieving personalized medical care for glioma patients (137). To the previously identified “3 Rs” (right patient, right time, and right drug), MI enables us to add a fourth “R”: right dosing.

Although MI offers several advantages, the use of this method in clinical research and practice currently remains at an early stage. Most MI probes are in the preclinical stage, while MI tracers targeting integrin α_V_β_3_, VEGF receptor, and IDH-mutation have been successfully translated to pilot studies in glioma patients. Another potential limitation is that most of these studies are based on the use of peptides, proteins, and even nanoparticles. Demand exists for designing small-molecule tracers that can cross the BBB, which generally hinders the use of MI in the case of LGG with relatively more intact BBB functionality compared to HGG.

Accelerating the clinical translation of MI to benefit patients with glioma will only be achieved with deft navigation of regulatory requirements and multi-center, international cooperation. Firstly, after the potential toxicity of MI probes has been tested in small-animal models, we recommend taking advantage of early exploratory Investigational New Drug studies (138). Due to the very low concentrations of injected tracers visualized on exquisitely sensitive MI platforms, this regulatory compliance strategy is more apt for MI research in an incurable disease such as GBM. Secondly, accrual of a sufficient number of patients into MI studies to make meaningful conclusions will require international multi-center clinical trials that are guided by uniform research protocols with built-in continual quality assessment and quality control.

## Author Contributions

DL and CP wrote the manuscript, under the supervision of LZ and ZC. Other authors participated in some discussions. All authors contributed to the article and approved the submitted version.

## Funding

This paper was partially sponsored by the Beijing Medical Research (2018-7), the National Natural Science Foundation of China Projects (81971668), Beijing Nova Program (xx2017017), Beijing Talents foundation, Clinical Scientist Supporting grant of Beijing Tiantan Hospital (YSP201902), as well as the funding from the Radiology Department at Stanford University.

## Conflict of Interest

The authors declare that the research was conducted in the absence of any commercial or financial relationships that could be construed as a potential conflict of interest.
